# Supplementary material for: A chronic Acinetobacter baumannii pneumonia model to study long-term virulence factors, antibiotic treatments, and polymicrobial infections
Source: Nat Commun. 2025 Aug 15;16:7617. doi: 10.1038/s41467-025-62655-4 (PMC12356904; doi:10.1038/s41467-025-62655-4)
Supplement: Supplementary file 1 — Supplementary Information [file 41467_2025_62655_MOESM1_ESM.pdf]

**SUPPLEMENTARY INFORMATION**

**Supplementary Tables**

**Table S1. MLST profiles for isolates screened in the long-term infection model.**

| Strain | Sequence type[a] | Profile | | | | | | |
|---|---|---|---|---|---|---|---|---|
| | | ***gltA*** | ***gyrB*** | ***gdhB*** | ***recA*** | ***cpn60*** | ***gpi*** | ***rpoD*** |
| **G636** | 208 | 1 | 3 | 3 | 2 | 2 | 97 | 3 |
| **G654** | 208 | 1 | 3 | 3 | 2 | 2 | 97 | 3 |
| **G803** | 1624 | 1 | 50 | 3 | 2 | 3 | 94 | 3 |
| ***Ab*19606** | 931 | 1 | 10 | 8 | 6 | 1 | 110 | 14 |
| ***Ab*17978** | 112 | 1 | 12 | 56 | 36 | 1 | 61 | 26 |

[a]MLST typing from PubMLST (1, 2).

**Table S2. Cytokine analysis at 4 h, 2 d, and 7 d post-intranasal infection with G636.**

| Cytokine[a,b] | WT | | | *tlr4* mutant | | | LOD[c] |
|---|---|---|---|---|---|---|---|
| **4 h** | $10^5$ G636 | $10^8$ G636 | Mock | $10^5$ G636 | $10^8$ G636 | Mock | |
| IL-23 | 23.26 (10.14) | 38.53 (6.36) | 53.80 (43.43) | 39.84 (20.38) | 32.61 (3.61) | 41.50 (27.96) | 2.55, 8.99 |
| IL-1α | 58.57 (18.27) | 323.11 (75.42)[#,$] | 2.5 (0.43) | 2.77 (0.47) | 429.19 (90.22)[#,$] | 1.49 (0.13) | 0.52, 0.51 |
| IFN-γ | 0.77 (0.36) | 5.13 (1.30)[#,$] | 0.00 (0.00) | 0.23 (0.23) | 4.34 (0.39)[#,$] | 0.00 (0.00) | 2.33, 0.78 |
| TNF-α | 4849.62 (2635.91) | 14888.73 (311.27)[#,$] | 108.38 (66.66) | 49.51 (6.47) | 12802.47 (3073.56)[#,$] | 12.82 (3.58) | 1.77, 1.11 |
| MCP-1 | 0.00 (0.00) | 65.73 (15.07)[#,$] | 0.00 (0.00) | 0.00 (0.00) | 65.57 (8.23)[#,$] | 0.00 (0.00) | 11.03, 15.06 |
| IL-12p70 | 0.00 (0.00) | 1.43 (1.43) | 0.00 (0.00) | 0.00 (0.00) | 0.00 (0.00) | 0.00 (0.00) | 3.91, 3.49 |
| IL-1β | 9.50 (1.95) | 46.26 (10.54)[#,$] | 0.00 (0.00) | 0.00 (0.00) | 56.58 (12.58)[#,$] | 0.00 (0.00) | 3.68, 3.75 |
| IL-10 | 0.00 (0.00) | 4.18 (4.18) | 0.00 (0.00) | 0.00 (0.00) | 2.04 (2.04) | 0.00 (0.00) | 8.03, 5.04 |
| IL-6 | 910.41 (172.71) | 7885.57 (1645.56)[#,$] | 18.19 (5.67) | 8.09 (2.69) | 6334.72 (1218.67)[#,$] | 2.70 (2.70) | 4.15, 2.71 |
| IL-27 | 0.00 (0.00) | 85.90 (13.54)[#,$] | 0.00 (0.00) | 0.00 (0.00) | 42.77 (24.83)[$] | 0.00 (0.00) | 64.39, 42.73 |
| IL-17A | 1.05 (0.74) | 8.36 (2.46)[#,$] | 0.00 (0.00) | 0.00 (0.00) | 4.66 (0.39)[#,$] | 0.00 (0.00) | 1.37, 1.60 |
| IFN-β | 0.00 (0.00) | 29.75 (17.23)[*,#,$] | 0.00 (0.00) | 0.00 (0.00) | 0.00 (0.00) | 0.00 (0.00) | 10.40, 51.80 |
| GM-CSF | 96.65 (13.37)[*,#] | 55.42 (7.19)[#,$] | 0.00 (0.00) | 0.00 (0.00) | 47.41 (6.96)[#,$] | 0.00 (0.00) | 14.80, 11.80 |
| **2 d** | | | | | | | |
| IL-23 | 76.58 (57.20) | 27.05 (8.76) | 44.09 (21.87) | 26.04 (11.37) | 11.35 (5.56) | 72.50 (36.30) | 2.55, 8.99 |
| IL-1α | 1.14 (0.19) | 157.28 (58.45)[*,#,$] | 1.13 (0.20) | 2.00 (0.50) | 25.68 (6.15) | 19.64 (18.02) | 0.52, 0.51 |
| IFN-γ | 2.20 (0.98) | 212.26 (54.96)[*,#,$] | 0.00 (0.00) | 3.70 (2.48) | 4.96 (2.48) | 0.00 (0.00) | 2.33, 0.78 |
| TNF-α | 1.85 (0.82) | 592.26 (154.11)[*,#,$] | 1.03 (0.60) | 19.79 (6.79) | 52.20 (8.86) | 5.76 (4.31) | 1.77, 1.11 |
| MCP-1 | 0.00 (0.00) | 160.30 (14.27)[*,#,$] | 0.00 (0.00) | 0.00 (0.00) | 33.52 (6.37)[#,$] | 0.00 (0.00) | 11.03, 15.06 |
| IL-12p70 | 0.00 (0.00) | 46.17 (17.63)[*,#,$] | 0.00 (0.00) | 0.00 (0.00) | 0.00 (0.00) | 0.00 (0.00) | 3.91, 3.49 |
| IL-1β | 1.06 (1.06) | 12.35 (2.80)[*,#,$] | 0.00 (0.00) | 0.00 (0.00) | 0.00 (0.00) | 0.00 (0.00) | 3.68, 3.75 |
| IL-10 | 0.00 (0.00) | 0.00 (0.00) | 0.00 (0.00) | 0.00 (0.00) | 0.00 (0.00) | 0.00 (0.00) | 8.03, 5.04 |
| IL-6 | 0.00 (0.00) | 1179.63 (323.29)[*,#,$] | 0.00 (0.00) | 0.00 (0.00) | 20.45 (3.83) | 4.29 (4.29) | 4.15, 2.71 |
| IL-27 | 0.00 (0.00) | 137.76 (81.43)[*,#,$] | 0.00 (0.00) | 0.00 (0.00) | 0.00 (0.00) | 0.00 (0.00) | 64.39, 42.73 |
| IL-17A | 0.42 (0.42) | 25.31 (10.22)[*,#,$] | 0.00 (0.00) | 0.00 (0.00) | 0.00 (0.00) | 0.00 (0.00) | 1.37, 1.60 |
| IFN-β | 2.87 (2.87) | 0.00 (0.00) | 0.00 (0.00) | 0.00 (0.00) | 0.00 (0.00) | 0.00 (0.00) | 10.40, 51.80 |
| GM-CSF | 0.00 (0.00) | 0.00 (0.00) | 0.00 (0.00) | 0.00 (0.00) | 0.00 (0.00) | 0.00 (0.00) | 14.80, 11.80 |
| **7 d** | | | | | | | |
| IL-23 | 41.43 (14.43) | 30.14 (19.51) | 20.66 (20.66) | 7.23 (4.70) | 29.77 (12.87) | 12.69 (12.69) | 2.55, 8.99 |
| IL-1α | 1.05 (0.18) | 41.50 (23.66) | 1.62 (0.92) | 5.54 (4.27) | 4.60 (2.50) | 1.79 (0.85) | 0.52, 0.51 |
| IFN-γ | 0.00 (0.00) | 376.35 (322.27) | 0.00 (0.00) | 0.00 (0.00) | 0.00 (0.00) | 0.00 (0.00) | 2.33, 0.78 |
| TNF-α | 0.00 (0.00) | 93.32 (36.14)[*,#,$] | 0.00 (0.00) | 3.17 (3.17) | 2.41 (1.54) | 0.00 (0.00) | 1.77, 1.11 |
| MCP-1 | 0.00 (0.00) | 14.83 (14.83) | 0.00 (0.00) | 0.00 (0.00) | 0.00 (0.00) | 0.00 (0.00) | 11.03, 15.06 |
| IL-12p70 | 0.00 (0.00) | 1.54 (1.54) | 0.00 (0.00) | 0.00 (0.00) | 0.00 (0.00) | 0.00 (0.00) | 3.91, 3.49 |
| IL-1β | 0.00 (0.00) | 1.43 (1.43) | 0.00 (0.00) | 0.00 (0.00) | 0.00 (0.00) | 0.00 (0.00) | 3.68, 3.75 |
| IL-10 | 0.00 (0.00) | 6.84 (6.84) | 0.00 (0.00) | 0.00 (0.00) | 0.00 (0.00) | 0.00 (0.00) | 8.03, 5.04 |
| IL-6 | 0.00 (0.00) | 232.41 (182.45) | 0.00 (0.00) | 5.19 (5.19) | 16.72 (16.72) | 0.00 (0.00) | 4.15, 2.71 |
| IL-27 | 0.00 (0.00) | 26.87 (26.87) | 0.00 (0.00) | 0.00 (0.00) | 0.00 (0.00) | 0.00 (0.00) | 64.39, 42.73 |
| IL-17A | 0.00 (0.00) | 13.67 (11.48) | 0.00 (0.00) | 0.00 (0.00) | 0.00 (0.00) | 0.00 (0.00) | 1.37, 1.60 |
| IFN-β | 0.00 (0.00) | 0.00 (0.00) | 0.00 (0.00) | 0.00 (0.00) | 0.00 (0.00) | 0.00 (0.00) | 10.40, 51.80 |
| GM-CSF | 0.00 (0.00) | 0.00 (0.00) | 0.00 (0.00) | 0.00 (0.00) | 0.00 (0.00) | 0.00 (0.00) | 14.80, 11.80 |

[a]Mean pg/ml (SEM) from two independent experiments at each timepoint is displayed.
[b]**P* < 0.05 relative to *tlr4* mutant at same inoculum; [#]*P* < 0.05 relative to mock in same mouse strain. [$]*P* < 0.05 relative to $10^5$ inoculum in same mouse strain. Two-way ANOVA, Tukey's test for multiple comparisons. Significant differences are also highlighted in green.
[c]Shown are LODs from the two independent experiments.

**Table S3. MICs for *A. baumannii* strains G636 and G654.**

| Antibiotic | G636 (Resistant/Sensitive) | G654 (Resistant/Sensitive) | Clinical Breakpoint[a] |
|---|---|---|---|
| **Imipenem** | >256 μg/ml (Resistant) | >256 μg/ml (Resistant) | 2 μg/ml |
| **Ampicillin** | >256 μg/ml (N/A) | >256 μg/ml (N/A) | N/A[c] |
| **Ciprofloxacin** | >256 μg/ml (Resistant) | >256 μg/ml (Resistant) | 1 μg/ml |
| **Levofloxacin** | 32 μg/ml (Resistant) | 128 μg/ml (Resistant) | 2 μg/ml |
| **Colistin** | 1 μg/ml (Intermediate)[b] | 8 μg/ml (Resistant) | 2 μg/ml[b] |
| **Polymyxin B** | 2 μg/ml (Intermediate)[b] | 4 μg/ml (Resistant) | 2 μg/ml[b] |
| **Tigecycline** | 2 μg/ml (N/A)[c] | 1 μg/ml (N/A)[c] | N/A[c] |
| **Gentamicin** | >256 μg/ml (Resistant) | 2-4 μg/ml (Sensitive) | 4 μg/ml |
| **Apramycin** | 16 μg/ml (N/A)[c] | 16 μg/ml (N/A)[c] | N/A[c] |

[a]Clinical breakpoints are according to the Clinical and Laboratory Standard Institute (CLSI) M100 Performance Standards for Antimicrobial Susceptibility Testing 30th Edition (3).

[b] A "sensitive" breakpoint is not available for colistin or polymyxin B from the CLSI. Strains with MICs of less than or equal to 2 μg/ml are considered to have "intermediate resistance."

[c]The clinical breakpoint has not been defined for ampicillin, tigecycline, and apramycin by the CLSI.

**Table S4. Plasmids and strains used in this study.**

| Plasmid or Strain | Description[a] | Source[b] |
|---|---|---|
| Plasmids | | |
| pEX18Tc | Precursor plasmid used for generation of pEX18Ap; Tet$^r$ | (4) |
| pKD4-Apr | Source for apramycin cassette for mutant generation; Apr$^r$ | (5) |
| pEX18Ap | Plasmid background used for generation of *A. baumannii* mutants; Apr$^r$ | This study |
| pEX18Ap::G636 *invL*KO | Plasmid used for mutation of *invL* in G636; Apr$^r$ | This study |
| pUC18T-miniTn7T-Apr | Vector used for genetic complementation at the mTn7 site; Apr$^r$ | (6) |
| pUC18T-miniTn7T-Apr::G636 *invL*KO comp | Plasmid used for complementation of the Δ*invL* mutant; Apr$^r$ | This study |
| PB-FLuc+GFPd2 | Plasmid source for *gfp* cassette; Amp$^r$ | [b] |
| pUC18T-miniTn7T-Apr::*gfpd2* | Expression vector; Apr$^r$ | This study |
| pRK2013 | Helper plasmid for mobilization of non-self-transmissible plasmids; Kan$^r$ | (7) |
| pTNS2 | T7 transposase expression vector; Amp$^r$ | (8) |
| Strains | | |
| *E. coli* | | |
| Stellar | *mrr-hsdRMS-mcrBC* and *mcrA*; Host strain for cloning | TaKaRa |
| HB101 | F- *mcrB mrr hsdS*20(rB- mB-) *recA*13 *leuB*6 *ara*-14 *proA*2 *lacY*1 *galK*2 *xyl*-5 *mtl*-1 *rpsL*20 *glnV*44 λ-; Host strain for pRK2013 | Promega |
| EC100D | F - mcrA Δ(*mrr-hsdRMS-mcrBC*) φ80*dlacZ*ΔM15 Δ*lacX*74 *recA*1 *endA*1 *araD*139 Δ(*ara*, *leu*)7697 *galU galK* λ- *rpsL nupG pir* +(DHFR); Host strain for pTNS2 | Fisher |
| *A. baumannii* | | |
| G636 | 2018 *A. baumannii* respiratory isolate (Strain 3689) | [c] |
| G636 Δ*invL* | G636 *invL* mutant | This study |
| G636 *invL*$^+$ | G636 *invL* mutant complemented | This study |
| G636 Δ*bap* | G636 *bap* mutant | This study |
| G636 Δ*ata* | G636 *ata* mutant | This study |
| G636 Δ*fhaBC* | G636 *fhaBC* mutant | This study |
| G636-*gfp* | G636 expressing *gfpd2* | This study |
| G654 | 2020 *A. baumannii* respiratory isolate (Strain 6919) | [c] |
| G803 | 2019 *A. baumannii* respiratory isolate (Strain ABM452) | [d] |
| *Ab*19606 | 1948 *A. baumannii* urinary isolate | (9) |
| *Ab*17978 | 1951 *A. baumannii* meningitis isolate | (10, 11) |
| *S. aureus* | | |

| | | |
|---|---|---|
| Newman | 1952 osteomyelitis isolate | (12) |
| *K. pneumoniae* | | |
| TOP52 | 2006 cystitis isolate | (13) |

[a]Tet, tetracycline; Apr, apramycin; Amp, ampicillin; Kan, kanamycin.
[b]PB-FLuc+GFPd2 was a gift from Jordan Green (Addgene plasmid # 127190; http://n2t.net/addgene:127190; RRID: Addgene_127190).
[c]Strains G636 and G654 were collected by the CDC-funded Georgia Emerging Infections Program's (EIP) Multi-site Gram-Negative Surveillance Initiative (MuGSI) and kindly provided by Sarah Satola.
[d]Strain G803 was collected in an Iranian hospital and provided to us by Masoumeh Douraghi of the Division of Microbiology in the Department of Pathobiology at Tehran University of Medical Sciences School of Public Health.

**Table S5. Primers used in this study.**

| Primer | Sequence |
|---|---|
| 5' pEX18 marker swap | ACACGGTGCCTGACTGCGTTAGC |
| 3' pEX18 marker swap | ATGGAAGCCGGCGGCACC |
| 5' Apr for pEX18Ap | GAGGTGCCGCCGGCTTCCATGATCCTCAGCCAATCGACTGGC |
| 3' Apr for pEX18Ap | AACGCAGTCAGGCACCGTGTGATTCCCTTTGTCAACAGCAATGG |
| 5' pEX18Tc | ATGCCTGCAGGTCGACTCTAGAGG |
| 3' pEX18Tc | GCAAGCTTGGCACTGGCCGT |
| 5' F1 G636 *invL*KO | ACGGCCAGTGCCAAGCTTGCGGCAATGTCTCAAATAAAAAATTTAACTC |
| 3' F1 G636 *invL*KO | TGAGATCCGCTATTATTACTTCCAG |
| 5' F2 G636 *invL*KO | AGTAATAATAGCGGATCTCATGCTTCTTTTTTTAGAGTTGTGTTCC |
| 3' F2 G636 *invL*KO | TAGAGTCGACCTGCAGGCATAAAATAACCGCATAGCCAGCTTGAGC |
| 5' G636 *fdeC*KO Comp | GCATGAGCTCACTAGTGGATCCGAGATTAAGACTTTACTTGGCATACACC |
| 3' G636 *fdeC*KO Comp-His6 v2 | TCAGTGGTGATGGTGATGATGATTACCATTTGAACAGTTTGGATCTATTCC |
| 5' F1 G636 *bap*KO | ACGGCCAGTGCCAAGCTTGCAGAAGCGGCTGGCAATGTCACG |
| 3' F1 G636 *bap*KO | TCAAGCACCGGTGCATACTGACC |
| 5' F2 G636 *bap*KO | CAGTATGCACCGGTGCTTGAGGTGGTAACACTACAATTCAGATTGACC |
| 3' F2 G636 *bap*KO | TAGAGTCGACCTGCAGGCATTCCATAAATGAATTTGCCATTTTCTTGAATCTG |
| 5' F1 G636 *ata*KO | ACGGCCAGTGCCAAGCTTGCTAAGTCGGTCTGGCTATTCGCC |
| 3' F1 G636 *ata*KO | TGATGACGTTGAGAAAAAAGCTAATGCAGG |
| 5' F2 G636 *ata*KO | CTTTTTTCTCAACGTCATCAAAAACTTCTCAGACAAATACCGAACTCAACG |
| 3' F2 G636 *ata*KO | TAGAGTCGACCTGCAGGCATCGTGATCAATTTCTTCTGTAAGCGAATCTTTTTGC |
| 5' F1 G636 *fhaBC*KO | ACGGCCAGTGCCAAGCTTGCTTAAAATTTTAAAGCAGTTTGATGAGCC |
| 3' F1 G636 *fhaBC*KO | CAGAATTGTACGTATAAGAACTTTATTTTACAC |
| 5' F2 G636 *fhaBC*KO | TTCTTATACGTACAATTCTGTAACAATGAAAAATGCACATGCGG |

| | |
|---|---|
| 3’ F2 G636 *fhaBC*KO | TAGAGTCGACCTGCAGGCATCTTTATTGGTACCCTGATTGCG |
| Tn7 linear Fwd-His6 | CATCATCACCATCACCACTGAAAGCTTGGGCCCGGTACCTC |
| Tn7 linear Rev | GGATCCACTAGTGAGCTCATGC |
| 5' d2EGFP for pUC18T-mTn7 | AGAAAGAGGAGAAATACTAGATGGTGAGCAAGGGCGAGG |
| 3' d2EGFP for pUC18T-mTn7 | GAGGTACCGGGCCCAAGCTTCTACACATTGATCCTAGCAGAAGC |
| 5' pUC18T-mTn7 for d2EGFP | AAGCTTGGGCCCGGTACCTCG |
| 3' pUC18T-mTn7 for d2EGFP | CTAGTATTTCTCCTCTTTCTCTAGTAATTGTTATCC |

**Supplementary Figures**

**Figure S1:**

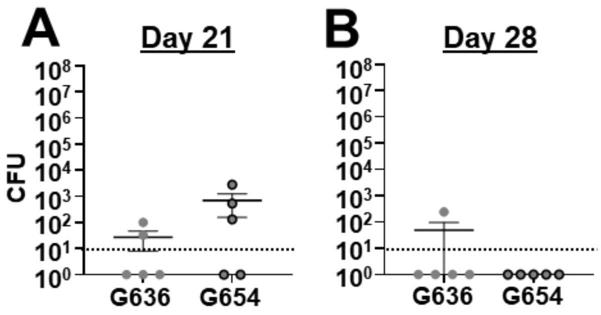


**Figure S1.** Modern respiratory isolates persist up to 3-4 weeks pi in the chronic respiratory infection model. Groups of female mice were intranasally inoculated with $10^5$ G636 or $10^5$ G654 and 21 (A) and 28 dpi (B), groups of 5 mice were sacrificed, and CFU were quantified in the lungs. The horizontal line represents the mean, and the SEM is indicated by error bars. The limit of detection (10 CFU) is indicated by the dashed line. Source data are provided as a Source Data file.

**Figure S2:**

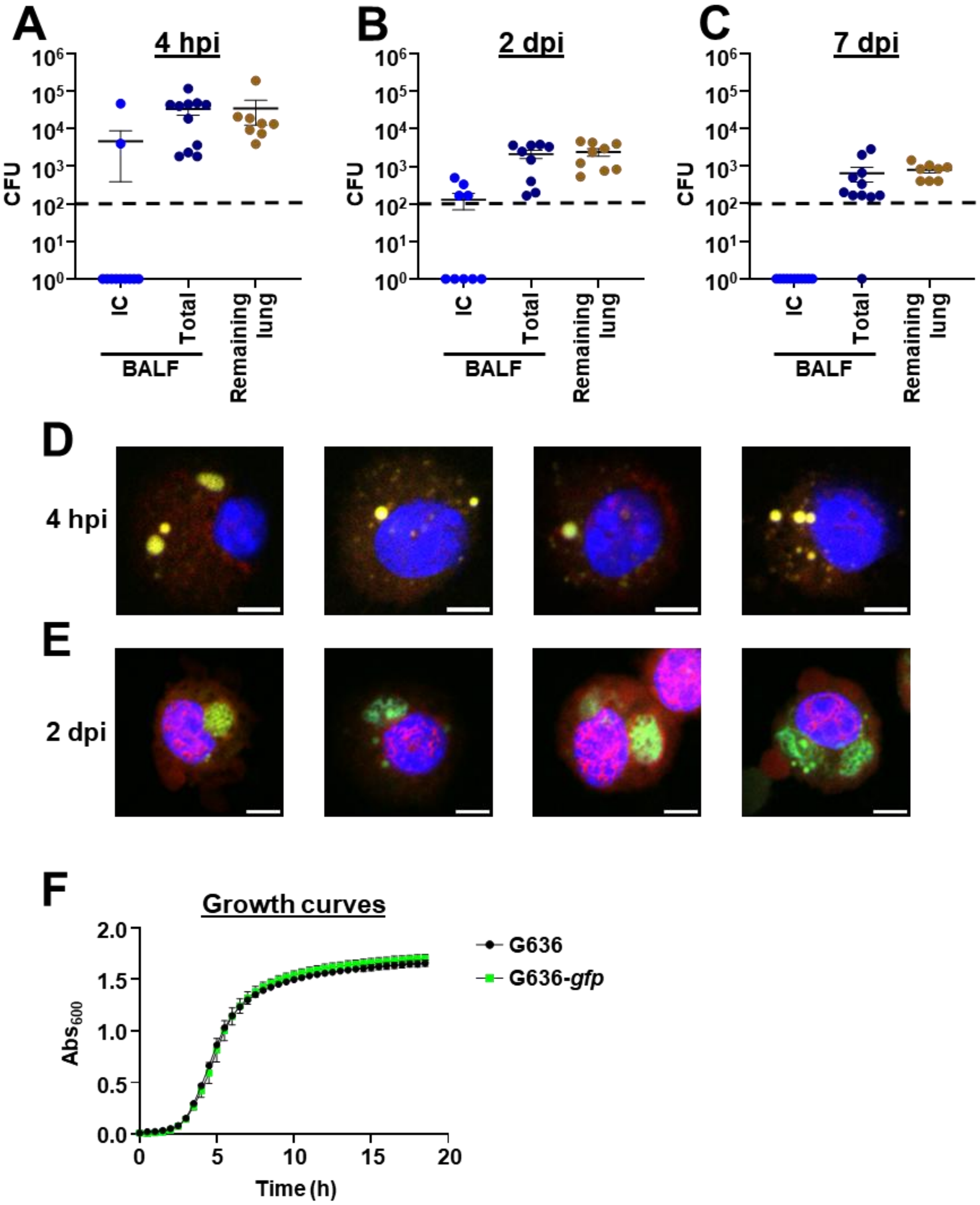


**Figure S2.** Intracellular *A. baumannii* are detectable in BALF at early timepoints in the chronic respiratory infection model. Groups of female C3H/HeJ (*tlr4* mutant) mice were intranasally inoculated with $10^5$ G636 expressing *gfp* (G636-*gfp*), and BALF was collected at 4 hpi (A), 2 dpi (B), and 7 dpi (C) and either treated with 50 µg/ml colistin or mock-treated. Bacterial CFU in the treated (intracellular; IC) and mock treated (total) BALF, as well as in the remaining lungs following BALF collection, were enumerated by serial dilution plating. The horizontal line represents the

mean, and the SEM is indicated by error bars. The limit of detection (100 CFU) is indicated by the dashed line. Shown are the results from at least two independent experiments. Female C3H/HeJ (*tlr4* mutant) mice were infected with G636-*gfp*, and, at these same timepoints, BALF was collected, and host cells were isolated and stained with DAPI (blue) and phalloidin (red). Intracellular bacteria were identified by microscopy at 4 hpi (D) and 2 dpi (E). Shown are representative images from independent samples from two biological replicates. Scale bar = 5 µm. (F) *In vitro* growth curves were performed to confirm similar growth of G636 and G636-*gfp*. $Abs_{600}$ readings were performed every 30 min for 18.5 h. Shown are the results of two independent experiments, the mean is indicated by filled circles, and SEM is indicated by error bars. Source data are provided as a Source Data file.

**Figure S3:**

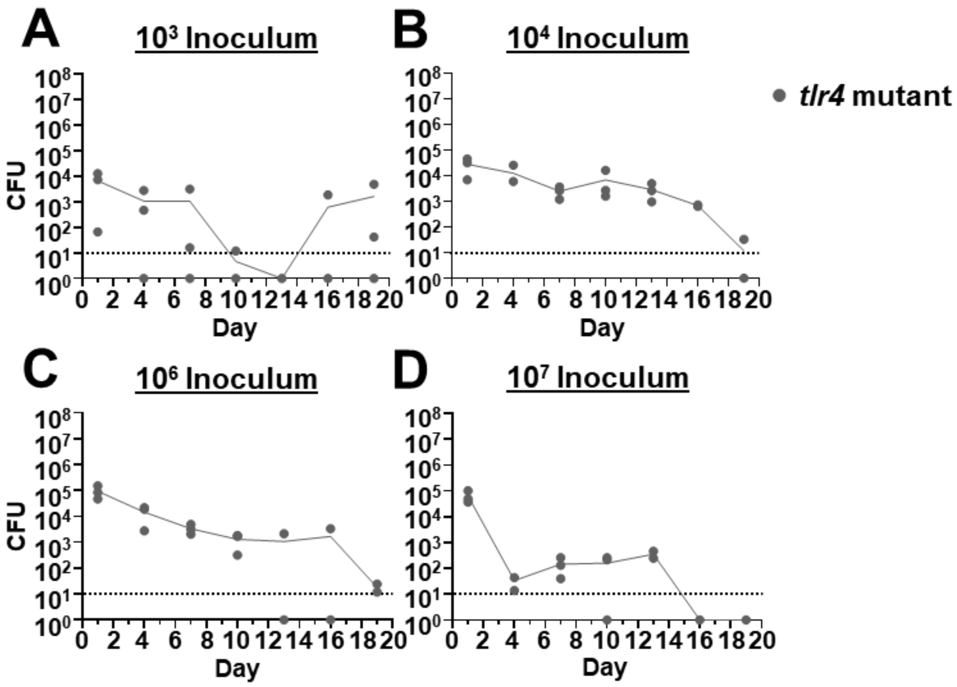


**Figure S3.** Outcomes of infection of C3H/HeJ with various inoculums. Groups of female C3H/HeJ (*tlr4* mutant) mice were intranasally inoculated with $10^3$ (A), $10^4$ (B), $10^6$ (C), or $10^7$ G636 (D). Beginning at 24 hpi, groups of mice were sacrificed every 3 days, and bacteria in the lungs were quantified. Each data point indicates and individual mouse, and the connecting line intersects each timepoint at the mean. The limit of detection (10 CFU) is indicated by the dashed line. Source data are provided as a Source Data file.

**Figure S4:**

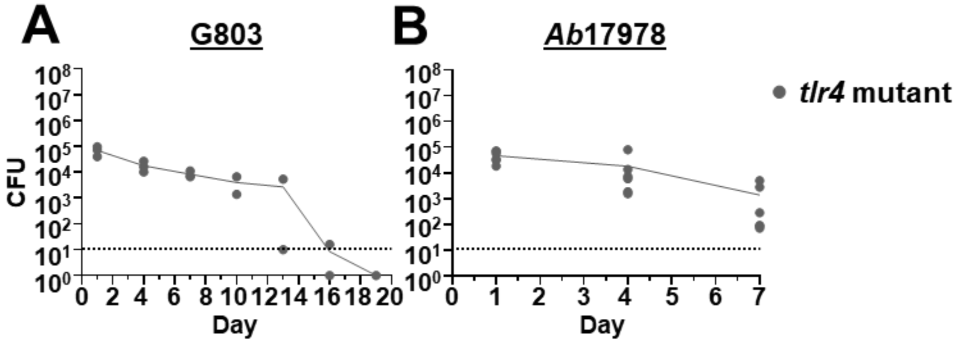


**Figure S4.** Outcomes of infection of C3H/HeJ with strains G803 and *Ab*17978. Groups of female C3H/HeJ (*tlr4* mutant) mice were intranasally inoculated with $10^5$ G803 (A) or $10^5$ *Ab*17978 (B). Beginning at 24 hpi, groups of mice were sacrificed every three days until day 19 for G803 and until day 7 for *Ab*17978, and bacteria in the lungs were quantified. Each data point indicates and individual mouse, and the connecting line intersects each timepoint at the mean. The limit of detection (10 CFU) is indicated by the dashed line. Source data are provided as a Source Data file.

**Fig. S5:**

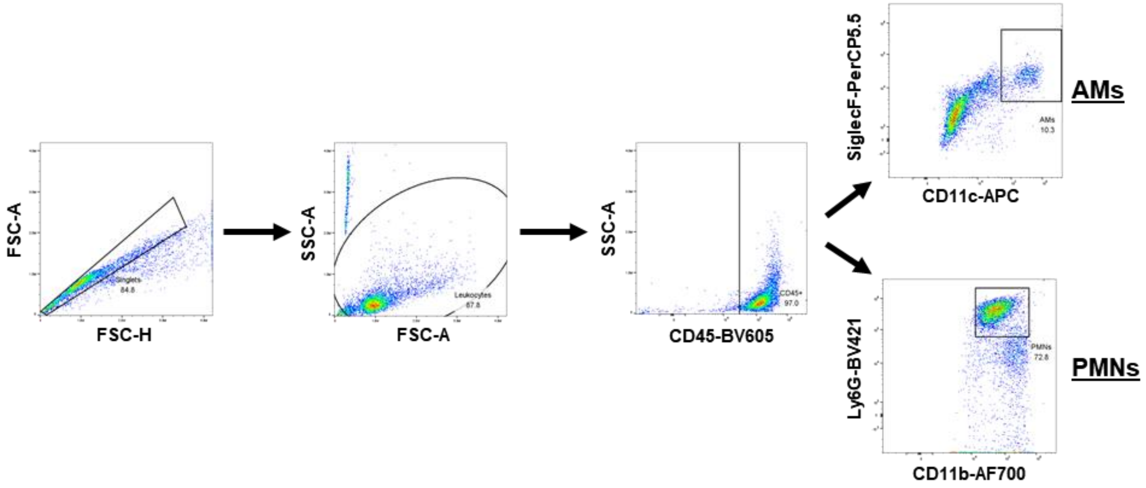


**Fig. S5.** Gating strategy for detection of AMs and PMNs in Figure 2. The gating strategy for flow cytometry to detect total AMs and PMNs in a representative mouse is shown. Forward scatter, FSC; Side scatter, SSC; BV605, brilliant violet 605; PerCP5.5, PerCP-cyanine5.5; allophycocyanin, APC; BV421, brilliant violet 421; AF700, Alexa Fluor 700.

**Figure S6:**

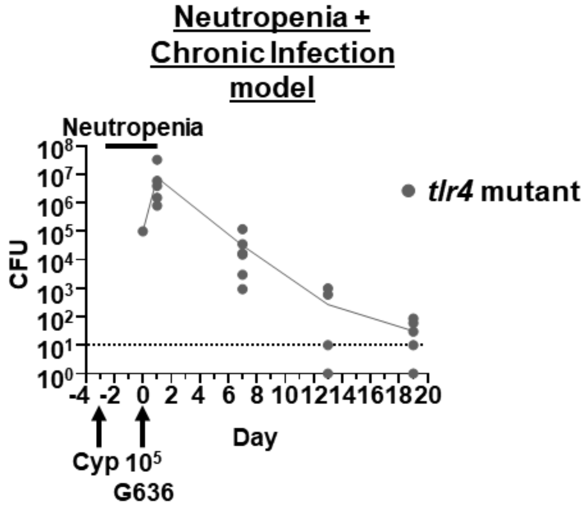


**Figure S6.** Impact of induced neutropenia early during the chronic infection model. Female C3H/HeJ (*tlr4* mutant) mice were treated with 300 mg/kg cyclophosphamide (Cyp) and, 3 days later, were infected with $10^5$ G636. The horizontal line labeled "neutropenia" indicates the established period of decreased neutrophils following cyclophosphamide treatment in mice. Beginning 1 dpi, every 6 days until day 19, groups of mice were sacrificed and bacterial CFU were quantified in the lungs. Shown are the pooled results from two independent experiments, each data point represents the mean, and the SEM is represented by error bars. The limit of detection (10 CFU) is indicated by the dashed line. Source data are provided as a Source Data file.

**Figure S7:**

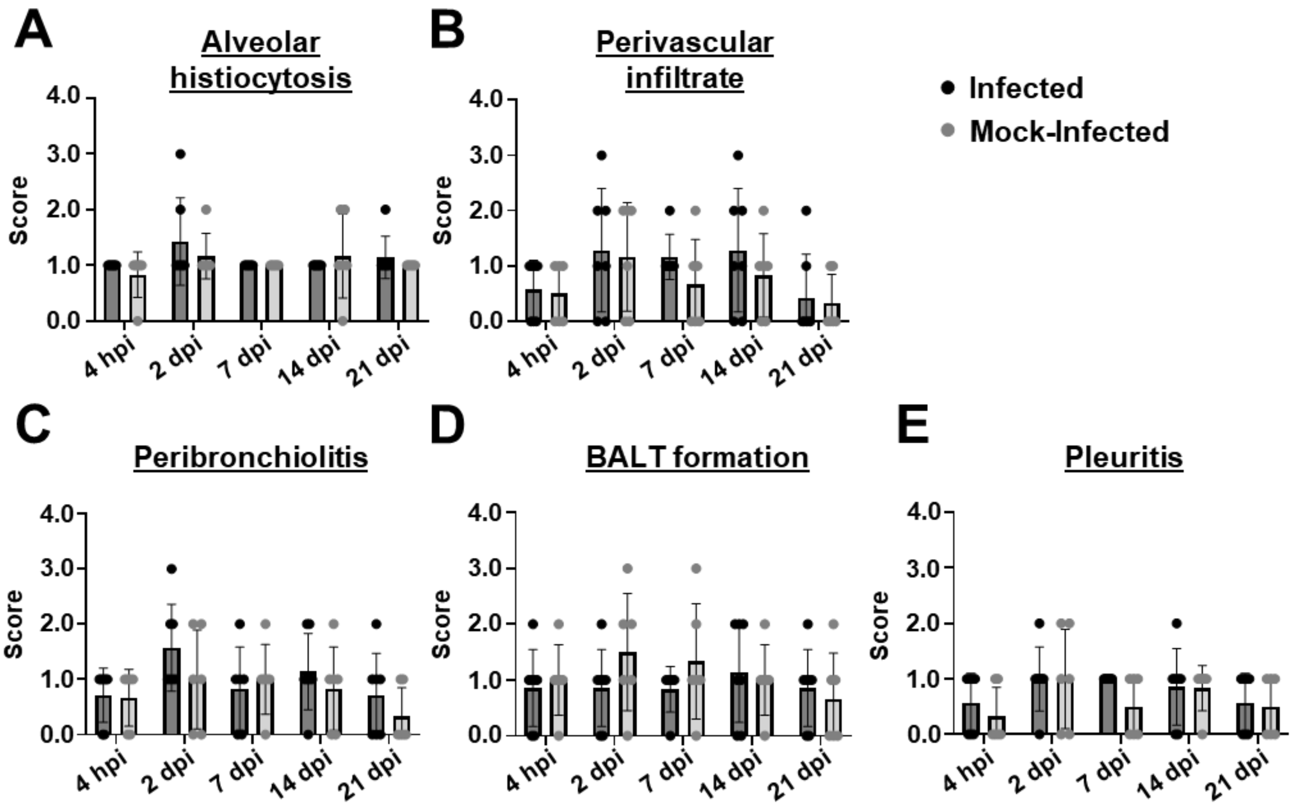


**Figure S7.** The chronic respiratory infection model results in no changes in alveolar histiocytosis, perivascular infiltrate, peribronchiolitis, BALT formation, or pleuritis. Groups of female C3H/HeJ (*tlr4* mutant) mice were inoculated with $10^5$ G636 or mock-inoculated with PBS, and at 4 hpi (A), 2 dpi (B), 7 dpi (C), 14 dpi (D), and 21 dpi (E), lungs slices were prepared, H&E stained, and scored for pathology. The bar represents the mean, each mouse is indicated by a dot, and the SEM is indicated by error bars. No values were significant; two-way analysis of variance (ANOVA), Bonferonni's test for multiple comparisons.

**Figure S8:**

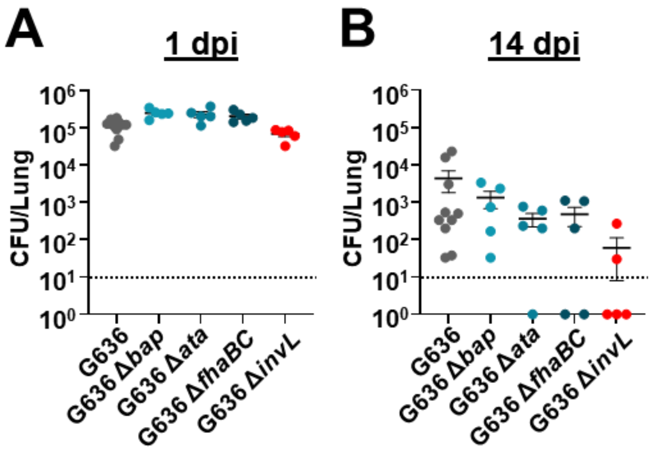


**Figure 8.** Testing of G636 adhesin mutants in the chronic respiratory infection model reveals a potential role for InvL in bacterial persistence. Groups of female C3H/HeJ (*tlr4* mutant) mice were intranasally inoculated with $10^5$ G636, G636 Δ*bap*, G636 Δ*ata*, G636 Δ*fhaBC*, and G636 Δ*invL*. 1 (A) and 14 (B) dpi, mice were sacrificed, and CFU in the lungs were quantified. Each data point represents an individual mouse, the horizontal line represents the mean, and the SEM is indicated by error bars. The limit of detection (10 CFU) is indicated by the dashed line. Shown are results from single experiments for each mutant strain. Source data are provided as a Source Data file.

**Figure S9:**

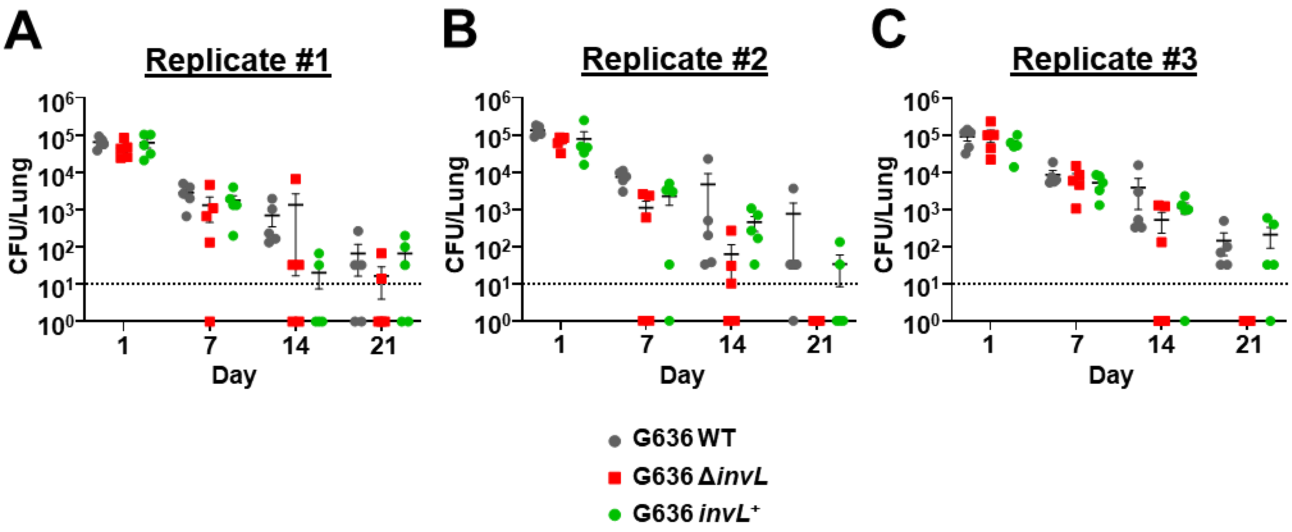


**Figure S9.** Independent replicates for Figure 4. C3H/HeJ (*tlr4* mutant) mice were infected with $10^5$ G636, G636 Δ*invL*, and G636 *invL*$^+$. Groups of female mice were then sacrificed at 1, 7, 14, and 21 dpi, and CFU in the lungs were quantified. Each data point represents an individual mouse, the horizontal line represents the mean, and the SEM is indicated by error bars. The limit of detection (10 CFU) is indicated by the dashed line. Source data are provided as a Source Data file.

**Figure S10:**

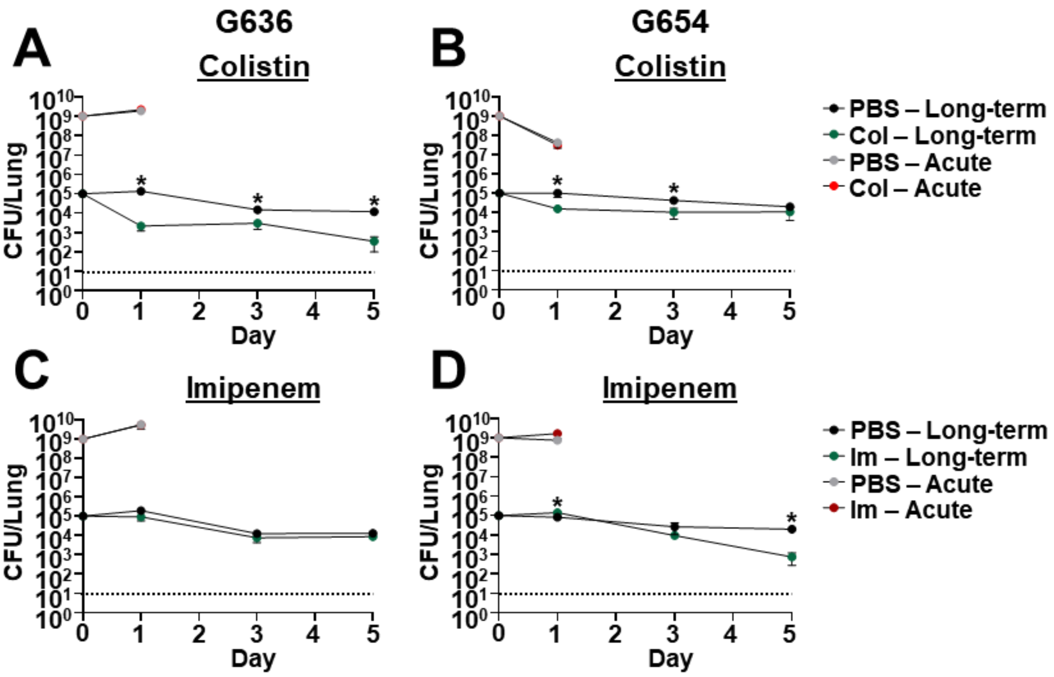


**Figure S10.** The chronic respiratory infection model can be used to study outcomes of antibiotic treatment. Groups of female C3H/HeJ (*tlr4* mutant) mice were infected with $10^5$ G636 (A, C) or $10^5$ G654 (B, D) and sacrificed at 1, 3, and 5 dpi (long-term). Additionally, groups of female C57Bl/6 mice were infected with $10^9$ G636 (A, C) or $10^9$ G654 (B, D) and sacrificed at 24 hpi (acute). Mice in both infection models were intraperitoneally treated with PBS or 5 mg/kg <u>col</u>istin (col) every 8 h (A, B) or PBS or 100 mg/kg <u>im</u>ipenem (im) every 12 h (C, D) with all treatments beginning 4 hpi. At each timepoint, CFU were quantified in the lungs. Shown are the results from at least two independent experiments for each group, each data point represents the mean, and the SEM is represented by error bars. The limit of detection (10 CFU) is indicated by the dashed line. $^*P < 0.05$; two-tailed Mann-Whitney *U* test. Source data and statistical test details are provided in the Source Data file.

**Figure S11:**

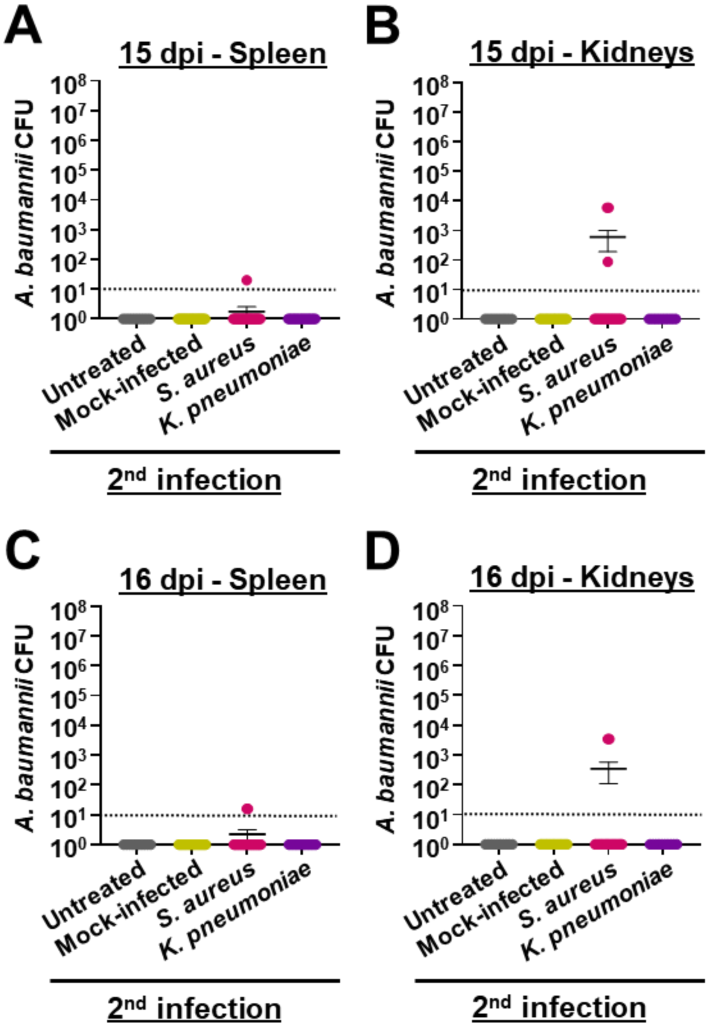


**Figure S11.** *S. aureus* secondary infection sometimes causes *A. baumannii* dissemination to the spleen and kidneys in the chronic respiratory infection model. Female C3H/HeJ (*tlr4* mutant) mice were intranasally inoculated with $10^5$ G636. At 14 days post-*A. baumannii* infection, groups of mice were either not inoculated (untreated), inoculated with PBS (mock-infected), infected with *S. aureus*, or infected with *K. pneumoniae*. Subsequently, on days 15 (A and B) and 16 (C and D) post-*A. baumannii* infection (1 and 2 days post-secondary infection), groups of mice were sacrificed, and *A. baumannii* CFU were quantified in the spleen (A and C), and kidneys (C and D). Each data point represents an individual mouse, the horizontal line represents the mean, and the SEM is indicated by error bars. The limit of detection (10 CFU) is indicated by the dashed line. Shown are results from at least 2 independent experiments for each group. Significant differences were not detected; Kruskal-Wallis *H* test with Dunn's test for multiple comparisons. Source data and statistical test details are provided in the Source Data file.

**Figure S12:**

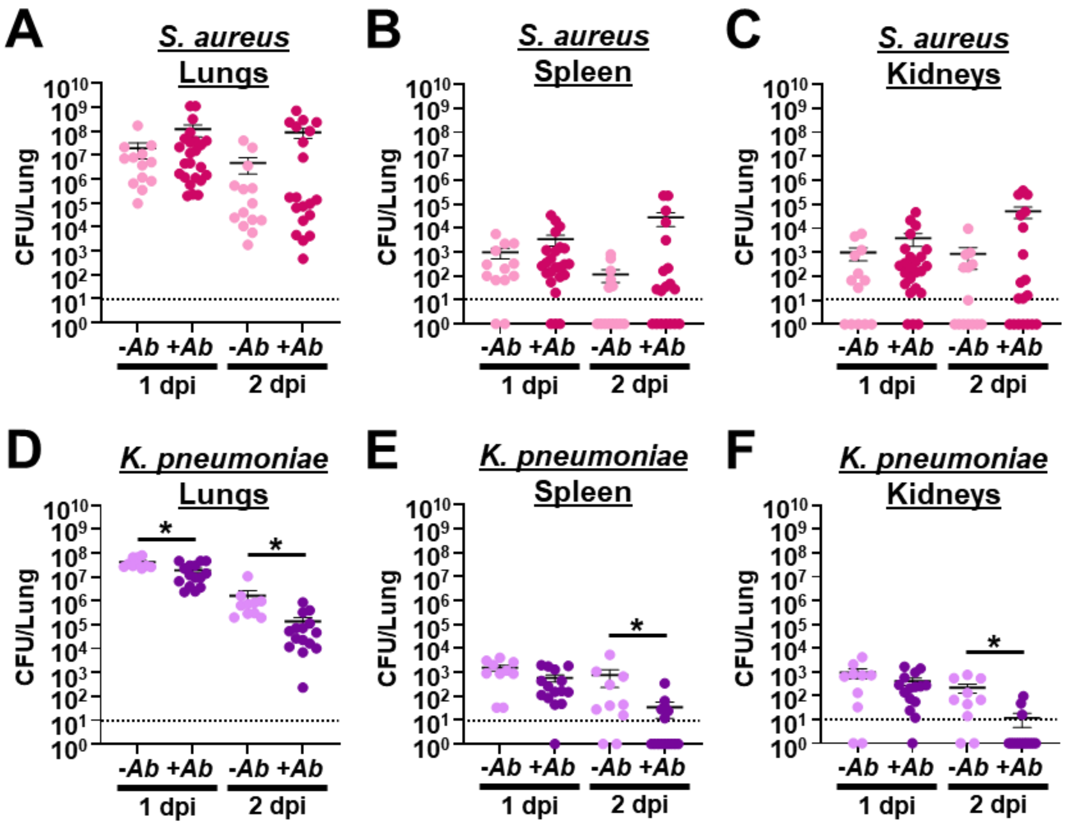


**Figure S12.** Ongoing *A. baumannii* pneumonia alters bacterial numbers following secondary infection with *S. aureus* and *K. pneumoniae*. Female C3H/HeJ (*tlr4* mutant) mice were either intranasally inoculated with $10^5$ G636 14 days prior to infection with *S. aureus* or *K. pneumoniae* (+*Ab*) or not infected prior to *S. aureus* or *K. pneumoniae* infection (-*Ab*). At 14 days post-*A. baumannii* infection, groups of mice were infected with *S. aureus* or *K. pneumoniae*. 1 and 2 dpi with *S. aureus* (A, B, and C) or *K. pneumoniae* (D, E, and F), mice were sacrificed, and these bacteria were quantified in the lung (A and D), spleen (B and E), and kidneys (C and F). Each data point represents an individual mouse, the horizontal line represents the mean, and the SEM is indicated by error bars. The limit of detection (10 CFU) is indicated by the dashed line. Shown are results from at least 2 independent experiments for each group. *$P < 0.05$; two-tailed Mann-Whitney *U* test. Source data and statistical test details are provided in the Source Data file.

**Supplementary Methods**

*Antibiotic susceptibility assays.*

MIC analyses were performed using a two-fold broth dilution microtiter assay similar to previously described protocols (14–16). Briefly, overnight cultures were sub-cultured at 0.05 $Abs_{600}$ and grown for 3 h shaking at 37 hpi. Mid-exponential growth phase cultures were then inoculated at 0.01 $Abs_{600}$ into a 96-well microtiter plate (Corning Inc, Corning, NY) containing two-fold decreasing dilutions of the indicated antibiotics. Plates were then incubated at 37°C with shaking for 24 h. The MIC was defined as less than 10% of the $Abs_{600}$ of an untreated control.

*Cytokine analysis.*

BALF was collected and centrifuged at 300 x *g* for 5 min. Supernatant containing cytokines was then collected and frozen at -20°C until the analysis was performed. Cytokine levels were determined using the LEGENDplex Mouse Inflammation Panel (13-plex) with V-bottom Plate (BioLegend) according to the manufacturer's instructions. Samples were read using an Aurora cytometer (Cytek Biosciences).

*Antibiotic protection assays.*

Antibiotic protection assays were performed as previously described (17). To determine the number of total and intracellular bacteria present in BALF from *A. baumannii* infected mice, two 500 µl aliquots of lavage fluid were centrifuged at 4100 x *g* for 5 min. Pelleted cells were resuspended in warm Dulbecco's Modified Eagle Medium (DMEM) (total bacteria) or DMEM with colistin (50 µg/mL) (intracellular bacteria) and incubated for 1h at 37°C. Samples were then washed three times with PBS and lysed with 500 µL of Triton X-100 (0.05%). CFUs were determined by serial dilutions of the bacterial suspensions. The remaining lungs following BALF collection were also homogenized, and CFUs were quantified by serial dilution plating.

*Cytospin of BALF cells.*

Cytospin of BALF cells was performed similar to previously described work (17). BALF samples were centrifugated at 300 x *g* for 5 min, and the pellets were resuspended in 1 mL Pharm Lyse Buffer (BD Biosciences) and incubated for 5 min on ice to lyse red blood cells. 9 mL of PBS was added to stop the lysis, viability was determined using Trypan Blue solution (Sigma-Aldrich, St. Louis, MO), and cells were counted using the TC20 Automated Cell Counter (Bio-Rad Laboratories, Hercules, CA). Samples were centrifugated at 300 x *g* for 6 min onto CytoPro Poly-L-Lysine Coated Microscope Slides (ELITechGroup Inc., Logan, UT) using a Cytospin Cytocentrifugue (Fisher Scientific, Hampton, NH). The slides were air-dried overnight at 4°C and fixed in 4% PFA for 30 min at room temperature. Samples were incubated with permeabilizing and blocking solution (PBS supplemented with 0.1% saponin, 0.5% bovine serum albumin, and 10% heat inactivated fetal bovine serum). Cells were stained with Alexa Fluor 555 Phalloidin (Cell Signaling Technology, Danvers, MA) and 4',6-Diamidino-2-phenylindole dihydrochloride (DAPI) solution (Invitrogen) for 1 h at 37°C. After staining, the samples were rinsed with washing solution [PBS supplemented with 0.1% saponin and 0.5% bovine serum albumin (BSA)], and then rinsed with water and mounted on a coverslip in ProLong Gold Antifade Mountant (Invitrogen).

*Confocal microscopy.*

Confocal microscopy was performed as previously described (17, 18). Microscopy slides were analyzed with a Zeiss LSM880 laser scanning confocal microscope (Carl Zeiss AG, Oberkochen, Germany) equipped with 405nm diode, 488nm Argon, 543nm HeNe, and 633nm HeNe lasers. A Plan-Apochromat 63X DIC objective and ZEN black 2.1 SP3 software were used for image acquisition. Images were analyzed using ImageJ software (National Institutes of Health, Bethesda, MD) (19).

**Supplementary References**


1. Jolley KA, Maiden MCJ. 2010. BIGSdb: Scalable analysis of bacterial genome variation at the population level. BMC Bioinformatics 11.

2. Maiden MCJ, Bygraves JA, Feil E, Morelli G, Russell JE, Urwin R, Zhang Q, Zhou J, Zurth K, Caugant DA, Feavers IM, Achtman M, Spratt BG. 1998. Multilocus sequence typing: a portable approach to the identification of clones within populations of pathogenic microorganisms. Proc Natl Acad Sci U S A 95:3140–3145.

3. M100 Performance Standards for Antimicrobial Susceptibility Testing A CLSI supplement for global application.

4. Hoang TT, Karkhoff-Schweizer RR, Kutchma AJ, Schweizer HP. 1998. A broad-host-range Flp-FRT recombination system for site-specific excision of chromosomally-located DNA sequences: application for isolation of unmarked *Pseudomonas aeruginosa* mutants. Gene 212:77–86.

5. Pontes MH, Groisman EA. 2019. Slow growth dictates non-heritable antibiotic resistance in *Salmonella enterica*. Sci Signal 12.

6. Ducas-Mowchun K, De Silva PM, Crisostomo L, Fernando DM, Chao TC, Pelka P, Schweizer HP, Kumar A. 2019. Next Generation of Tn 7-Based Single-Copy Insertion Elements for Use in Multi- and Pan-Drug-Resistant Strains of *Acinetobacter baumannii*. Appl Environ Microbiol 85.

7. Figurski DH, Helinski DR. 1979. Replication of an origin-containing derivative of plasmid RK2 dependent on a plasmid function provided in trans. Proc Natl Acad Sci U S A 76:1648–1652.

8. Choi KH, Gaynor JB, White KG, Lopez C, Bosio CM, Karkhoff-Schweizer RAR, Schweizer HP. 2005. A Tn7-based broad-range bacterial cloning and expression system. Nat Methods 2:443–448.

9. HUGH R, REESE R. 1968. A comparison of 120 strains of *Bacterium anitratum* Schaub and Hauber with the type strain of this species. Int J Syst Evol Microbiol 18:207–229.

10. PIECHAUD M, SECOND L. 1951. [Studies of 26 strains of Moraxella *Iwoffi*]. Ann Inst Pasteur (Paris) 80:97–9.

11. Baumann P, Doudoroff M, Stanier RY. 1968. A study of the Moraxella group. II. Oxidative-negative species (genus Acinetobacter). J Bacteriol 95:1520–1541.

12. DUTHIE ES, LORENZ LL. 1952. Staphylococcal coagulase; mode of action and antigenicity. J Gen Microbiol 6:95–107.

13. Rosen DA, Hooton TM, Stamm WE, Humphrey PA, Hultgren SJ. 2007. Detection of Intracellular Bacterial Communities in Human Urinary Tract Infection. PLoS Med 4:1949–1958.

14. McGuffey JC, Jackson-Litteken CD, Venanzio G Di, Zimmer AA, Lewis JM, Distel JS, Kim KQ, Zaher HS, Alfonzo J, Scott NE, Feldman MF. 2023. The tRNA methyltransferase TrmB is critical for *Acinetobacter baumannii* stress responses and pulmonary infection. mBio 14.

15. Leus I V., Adamiak J, Trinh AN, Smith RD, Smith L, Richardson S, Ernst RK, Zgurskaya HI. 2020. Inactivation of AdeABC and AdeIJK efflux pumps elicits specific nonoverlapping transcriptional and phenotypic responses in *Acinetobacter baumannii*. Mol Microbiol 114:1049–1065.

16. Leus I V., Weeks JW, Bonifay V, Smith L, Richardson S, Zgurskaya HI. 2018. Substrate Specificities and Efflux Efficiencies of RND Efflux Pumps of *Acinetobacter baumannii*. J Bacteriol 200.

17. Distel JS, Di Venanzio G, Mackel JJ, Rosen DA, Feldman MF. 2023. Replicative *Acinetobacter baumannii* strains interfere with phagosomal maturation by modulating the vacuolar pH. PLoS Pathog 19.

18. Jackson-Litteken CD, Venanzio G Di, Le NH, Scott NE, Djahanschiri B, Distel JS, Pardue EJ, Ebersberger I, Feldman MF. 2022. InvL, an Invasin-Like Adhesin, Is a Type II Secretion System Substrate Required for *Acinetobacter baumannii* Uropathogenesis. mBio 13.

19. Schneider CA, Rasband WS, Eliceiri KW. 2012. NIH Image to ImageJ: 25 years of image analysis. Nat Methods 9:671–675.

120. Leus I V., Adamiak J, Trinh AN, Smith RD, Smith L, Richardson S, Ernst RK, Zgurskaya HI. 2020. Inactivation of AdeABC and AdeIJK efflux pumps elicits specific nonoverlapping transcriptional and phenotypic responses in Acinetobacter baumannii. Mol Microbiol 114:1049–1065.

121. Leus I V., Weeks JW, Bonifay V, Smith L, Richardson S, Zgurskaya HI. 2018. Substrate Specificities and Efflux Efficiencies of RND Efflux Pumps of Acinetobacter baumannii. J Bacteriol 200.

122. Jolley KA, Maiden MCJ. 2010. BIGSdb: Scalable analysis of bacterial genome variation at the population level. BMC Bioinformatics 11.

123. Maiden MCJ, Bygraves JA, Feil E, Morelli G, Russell JE, Urwin R, Zhang Q, Zhou J, Zurth K, Caugant DA, Feavers IM, Achtman M, Spratt BG. 1998. Multilocus sequence typing: a portable approach to the identification of clones within populations of pathogenic microorganisms. Proc Natl Acad Sci U S A 95:3140–3145.

124. M100 Performance Standards for Antimicrobial Susceptibility Testing A CLSI supplement for global application.

125. Figurski DH, Helinski DR. 1979. Replication of an origin-containing derivative of plasmid RK2 dependent on a plasmid function provided in trans. Proc Natl Acad Sci U S A 76:1648–1652.

126. Choi KH, Gaynor JB, White KG, Lopez C, Bosio CM, Karkhoff-Schweizer RAR, Schweizer HP. 2005. A Tn7-based broad-range bacterial cloning and expression system. Nat Methods 2:443–448.

127. HUGH R, REESE R. 1968. A comparison of 120 strains of Bacterium anitratum Schaub and Hauber with the type strain of this species. Int J Syst Evol Microbiol 18:207–229.

128. PIECHAUD M, SECOND L. 1951. [Studies of 26 strains of Moraxella lwoffi]. Ann Inst Pasteur (Paris) 80:97–9.

129. Baumann P, Doudoroff M, Stanier RY. 1968. A study of the Moraxella group. II. Oxidative-negative species (genus Acinetobacter). J Bacteriol 95:1520–1541.

130. DUTHIE ES, LORENZ LL. 1952. Staphylococcal coagulase; mode of action and antigenicity. J Gen Microbiol 6:95–107.

131. Rosen DA, Hooton TM, Stamm WE, Humphrey PA, Hultgren SJ. 2007. Detection of Intracellular Bacterial Communities in Human Urinary Tract Infection. PLoS Med 4:1949–1958.
